# A prospective randomized comparative trial evaluating postoperative nutritional intervention in patients with oral cancer

**DOI:** 10.1038/s41598-022-18292-8

**Published:** 2022-08-20

**Authors:** Hannah Nett, Julius Steegmann, Birgit Tollkühn-Prott, Frank Hölzle, Ali Modabber

**Affiliations:** 1 Department of Oral and Maxillofacial Surgery, RWTH Aachen University, Pauwelsstraße 30, 52074 Aachen, Germany; 2 Medical Clinic III – Department of Gastroenterology, Metabolic Disorders and Internal Intensive Medicine, RWTH Aachen University, Pauwelsstraße 30, 52074 Aachen, Germany

**Keywords:** Diseases, Gastroenterology, Health care, Oncology, Risk factors

## Abstract

Extensive surgical treatment of oral cancer results in significant deterioration of nutritional status with concomitant increased nutrient requirements. The consequences are an elevated risk of postoperative complaints as well as morbidity and mortality. The aim of this study was to investigate an additional postoperative nutritional intervention through professional nutritional advice and nutritional supplementation in patients with oral cancer for at least six months. 62 patients with oral cancer in the department of oral and maxillofacial surgery were randomized into two groups. The intervention group received nutritional supplements, protein-rich, high-fiber diet and care by a professional nutritionist in addition to the standard treatment. The control group received only the standard treatment. Statistical analysis includes the evaluation of means and standard deviations as well as the calculation of *p* values with a significance level of 0.05. A deficiency of protein, albumin, vitamin D, zinc and iron was noticed in both groups immediately after surgery. Patients in the intervention group recorded significantly less weight loss (pT2 = 0.0031, pT4 = 0.0424), a more stable BMI (pT2 = 0.0496), better values for albumin (pT2 = 0.0265), vitamin A (pT3 = 0.0248, pT4 = 0.0007) and calcium (pT3 = 0.0362) during the follow-ups. The patients in the intervention group showed significantly fewer digestive problems (*p* = 0.0062) and muscular complaints (*p* = 0.0448). They showed better eating habits (*p* = 0.0348) and were capable of more physical activity (*p* = 0.0045) than patients in the control group. Patients with oral cancer can have a benefit from postoperative nutritional intervention. Early screening, appropriate care by a nutritionist and supplementation with vitamin D, zinc, calcium and protein-rich food are recommended.

## Introduction

The proportion of HNC (Head and Neck Cancer) patients already having weight loss at the time of the diagnosis is reported in the literature up to 50%^1,2^ and illustrates the strong need for treatment of malnutrition in the multidisciplinary therapy of oral cancer. In particular, surgical and radiotherapeutic therapy measures result in an increased need for macro- and micronutrients in the postoperative period^3^.

Especially tumor treatments with oral involvement favor pre- and postoperative difficulties in eating. The often reduced uptake of nutrients and exposure to anorexic mediators, catabolic hormones and proinflammatory cytokines lead to an immediately postoperative catabolic metabolic state with a simultaneous increase in energy and nutrient consumption. The catabolic metabolism indicates lipolysis and gluconeogenesis, protein degradation and sarcopenia^4–6^. In the subsequent inpatient rehabilitation, the anabolic phase promotes the patient`s increased nutritional need.

Not infrequently, the reduced food intake combined with increased requirements leads to massive weight and muscle loss and to a decrease in physical resources and promote morbidity and mortality^7^. Cancer-related weight loss is associated with reduced tolerance and response to anticancer therapy and indicates a prolonged hospital stay^8^. In addition, malnutrition leads to poorer rehabilitation results, so optimization should be aimed at^9^.

For better immune defense and wound healing, cardiovascular resistance and cell replication should be promoted. This requires countermeasures in the event of a nutritional deficiency and a risk of malnutrition. Consequently, a necessary integration of patients into a professional nutritional care by specialized personal represents an often neglected aspect in the context of therapy and rehabilitation management, since physicians and nursing staff often lack necessary knowledge in this area^10^. Corresponding recommendations should be based on randomized, clinical studies with a sufficiently long follow-up. Unfortunately, these are rare as they require extensive resources. Studies in guidelines focus in particular on radiation or chemotherapy of patients, less on patients with reconstructive surgical measures, so they can only be quoted for comparison to a limited extent.

Therefore, the aim of the study was to investigate an additional postoperative nutritional intervention in the form of professional nutritional counseling and nutrient supplementation in patients with oral cancer over a six-month period in a randomized study design.

## Material and methods

**Figure 1:**
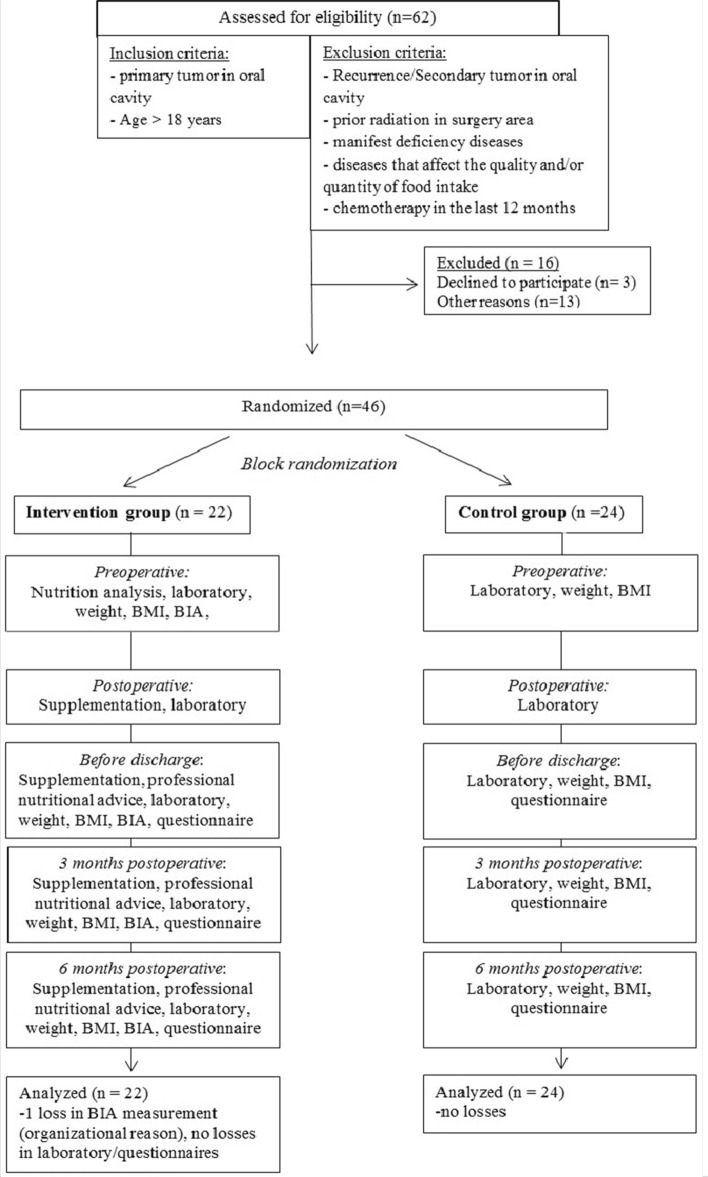
Summary of patient flow diagram.

The present randomized clinical trial was approved by the local ethical committee Ethik- Kommission an der medizinischen Fakultät der RWTH Aachen (registration number: EK 253/18) on 18/11/2018 and was registered at the German Clinical Trials Register DRKS (registration number: DRKS00016020, URL: https://www.drks.de/drks_web/navigate.do?navigationId=trial.HTML&TRIAL_ID=DRKS00016020) on 22/11/2018. The authors make sure that the described work was performed in accordance with the Code of Ethics of the World Medical Association (Declaration of Helsinki) for human experiments. Figure 1 illustrates the course of studies.

After institutional approval and informed consents, a total of 46 patients with oral cancer from the Clinic for Oral and Maxillofacial Surgery were randomized into two groups. One group received a postoperative additional nutritional support in form of nutritional supplementation and care by a nutritional assistant. The second group received only standard nutrition und regular checkups.

### Eligibility criteria

The exclusion criteria for this study were: Recurrences or secondary tumors in the oral cavity, head and neck cancer without oral involvement, prior radiation therapy in the operating area, chemotherapy in the last 12 months, manifest deficiency diseases or diseases that influence the quality and/or quantity of food intake, massive cognitive deficits and age < 18 years. The presence of previous nutritional therapy was inquired at the study enrolment and history taking. There was no study patient with previous nutritional treatment.

### Study procedure

#### Methods

66 laboratory parameters from the areas of hematology, coagulation, clinical chemistry, vitamins, trace elements, iron and thyroid metabolism were determined in both groups at five measurement time points (preoperative, one day postoperative, before discharge, 3 and 6 months after surgery). Weight and BMI were collected at four time points (preoperative, one day postoperative, before discharge, 3 and 6 months postoperative). To assess postoperative discomfort and patient- specific aspects, both groups documented comparable questionnaires before discharge and after three and six months postoperative.

#### Intervention and study process

After patient information and exclusion of contraindications, preoperative blood sampling and weight determinations were performed in both study groups. The intervention patients also received initial nutritional counseling.

After surgical tumor ablation with simultaneous microvascular defect coverage, the intervention patients were consulted by a nutritionist in the intensive care unit on the first postoperative day. The food supply was controlled and optimized immediately postoperatively via the gastric tube or if already preoperatively planned via a PEG system. Parenteral nutrition, vitamins and trace elements were supplemented if necessary. Depending on the state of swelling, swallowing function and the type and extent of the resection, the diet was oralized as soon as possible. The nourishing of the control group patients by gastric tube or PEG was controlled only by the staff of the intensive care unit. There was no additional supplementation of vitamins or trace elements and no additional professional advice from a nutritionist.

The intervention patients continued to receive 30-min professional nutritional advice and BIA measurement at four times (preoperative, before discharge, 3 and 6 months after surgery). Dietitians worked with patients to create a nutrition plan and sensitized patients to high-protein, high-fiber diets. They also motivated the patients to increase their daily physical activity.

Blood samples were taken to determine parameters and questionnaires were given to evaluate postoperative complaints and patient-specific aspects in both groups at the measurement times before discharge and three and six months postoperative.

From the first postoperative day, the intervention patients received additional high-fiber, high-protein food and, based on the laboratory parameters, individual micronutrient supplementation.

The self-created questionnaires are used to evaluate baseline characteristics, postoperative complaints, and nutritional aspects, as well as to evaluate the patients' nutritional counseling. They are not validated and have not been used in any other study before. However, the questionnaires were based on nutrition scores (Subjective Global Assessment (SGA), Nutritional Risk Core (NRS 2000)) and questions from other studies^2,11–13^. The responses of the patient-specific aspects were scored on a scale from 0 (low) to 10 (high).

#### Supplementation

In addition to the standard arranged food, the intervention patients received 1 bottle of the protein-containing diet (Fortimel Extra 200 ml, high energy protein rich) per day. It is a high-calorie, fiber-containing, balanced drinking food with an energy amount of 400 kcal, 18 g protein and 5 g soluble fiber. Substitution is done in the inpatient period. In the post-inpatient period, the prescription was made at reduced protein and albumin levels. If there was a lack of micronutrients, substitution was made with multivitamin preparations A–Z from Doppelherz (0-1-0), Decristol 20,000 1 × week, vitamin B12 1000 µg (0-1-0), zinc tablets 40 mg (1–0-0), calcium tablets 1000 mg (0–1-0) and/or iron tablets 100 mg (0-1-0). The supplementation of vitamins and trace elements was based on the laboratory values. During the inpatient stay, intake was monitored by the study investigators, while supplementation in the post-inpatient period was determined by the questionnaires.

#### Outcomes

The laboratory and BIA measurements were defined as primary endpoints. The secondary endpoints were set as the questionnaire results on postoperative discomfort and patient-specific aspects.

#### Sample size

The existing literature on postoperative nutritional intervention on patients with head and neck cancer was overviewed to derive a sensitive sample size range. The case size was set at 50 (25/25) patients and is comparable to similar prospective randomized studies on the topic^14–17^.

The statistical program G*Power (Heinrich Heine University, Düsseldorf, Germany) was used for sample calculation. With an alpha value of 0.05 (two-tailed) and a statistical power of 80%, there was a required sample size of 44 patients (22/22) to conduct this study to reject the null hypothesis concerning the effect of the additional nutritional intervention of 80% power and a 95% confidence interval.

The inclusion and exclusion criteria chosen allowed the formation of homogeneous study groups. Comparable studies did not differentiate between primary tumors and recurrences/secondary tumors and included all subgroups of head and neck tumor patients without subgroup analysis^14–17^.

#### Randomization

The group allocation was done by block randomization. Study patients whose initial surgical therapies took place on a Monday or Tuesday were included in the control group. Study patients whose operations took place on a Wednesday, Thursday or Friday were included in the intervention group. Patients did not know which group they would be assigned to at the time of randomization.

### Data processing and statistics

The statistical evaluation and the graph creation were conducted using GraphPad Prism version 9.0.0 for Windows, GraphPad Software, San Diego, California, USA, www.graphpad.com. The data were checked for normality using the D’Agostino-Pearson normality test.

For statistical analysis between the two groups, the two-tailed t-test was used for normally distributed values and the Mann–Whitney- test was used for nonparametric distributed values. For analysis within the groups, the one-sided t-test was performed for normally distributed values and the Mann–Whitney-test for nonparametric distributed values. The analysis of nominal distributions was carried out using Fisher's Exact test for low values and using the Chi-Square test for larger values.

A *p*-value of ≤ 0.05 was set as the cut-off for significance. Mean values ± standard deviations were determined for all data.

## Results

The recruitment, patient analysis during hospital stay and the follow-ups were performed between November 2018 and July 2020. The baseline characteristics in Table 1 show no significant differences between the intervention and control group. The information was determined using the patient files of the CGM Medico program and the questionnaires created.

**Table 1:** Baseline characteristics.

Baseline Characteristics	(Measurement time T0)				
Variable	Intervention group		Control group		*P* Value
	M	± SD	M	± SD	
Age	63.55	13.61	61.67	14.61	0.6511
Male	10 (45.46%)		15 (62.50%)		0.3746
Female	12 (54.55%)		9 (37.50%)		
Weight preoperative	76.56	22.66	74.75	18.26	0.7684
BMI preoperative	26.84	6.06	24.98	4.80	0.2586
Nikotin	17 (77.27%)		16 (66.67%)		0.5207
Alcohol	3 (13.63%)		3 (12.50%)		> 0.999
Educational qualification	3.59	2.96	4.35	3.45	0.1250
ASA Classification	2.41	0.67	2.25	0.53	0.3787
**Tumor entity**					0.1793
Squamosa cell carcinoma	18 (81.82%)		23 (95.83%)		
Other entities	4 (18.18%)		1 (4.17%)		
**Tumor size**					0.9374
T1	6 (27.27%)		6 (25.00%)		
T2	10 (45.46%)		10 (41.67%)		
T3	3 (13.64%)		5 (20.83%)		
T4	3 (13.64%)		3 (12.50%)		
Adjuvant Radiotherapy/Chemotherapy	7 (31.81%)		10 (41.66%)		0.5520
**Tumor localization**					0.2625
Tongue	9 (40.91%)		3 (12.50%)		
Base oft he mouth	2 (9.09%)		5 (20.33%)		
Planum bukkale	1 (4.55%)		4 (16.66%)		
Alveolar process	5 (22.72%)		7 (29.17%)		
Hard palate	1 (4.55%)		0 (0.00%)		
Soft palate	3 (13.64%)		3 (12.50%)		
Tonsills	1 (4.55%)		2 (8.33%)		
PEG (enteral nutrition system)	5 (22.73%)		7 (29.17%)		0.7419
Pain preoperative	2.18	2.02	2.13	1.87	0.8804
Nutritional behavior	2.55	1.74	2.33	1.63	0.6577
Physical activity	4.46	1.87	4.88	1.96	0.4610

M = mean, SD = standard deviation, PEG = percutaneous endoscopic gastroscopy.

Table 2 shows the measurement parameters of the BIA (intervention group only) (mean values, standard deviations and p-values between T0 and the further measurement time points T2,T3 and T4).The measured values of the important nutrition-specific BIA parameters phase angle, lean mass, and body cell mass (BCM) were maintained within the reference range despite significant reductions. The ECM/BCM index associated with postoperative mean values > 1 the risk for malnutrition in patients with oral cancers.

**Table 2:** BIA at measurement times T0 (preoperative), T2 (before discharge), T3 (3 months postoperative) and T4 (6 months postoperative) in intervention group.

Parameters	T0	T2	*P* Value T0/T2	T3	*P* Value T0/T3	T4	*P* Value T0/T4
	M ± SD	M ± SD		M ± SD		M ± SD	
Phase angle (I)	5.35 (0.97)	4.82 (1.06)	*0.0014*	5.00 (1.13)	*0.0022*	5.21 (1.15)	0.1628
Body water (I)	39.61(11.91)	40.11 (11.29)	0.4559	38.81 (11.61)	0.1419	38.84 (11.52)	0.1353
Lean mass (I)	54.10 (16.32)	54.22 (16.10)	0.3635	53.01 (15.87)	0.1419	52.98 (15.95)	0.1263
ECM(I)	27.79 (7.88)	29.70 (8.897)	0.0614	28.28 (7.59)	0.1744	27.61 (7.65)	0.4717
BCM(I)	26.35 (9.30)	24.56 (9.26)	< *0.0001*	24.62 (9.69)	< *0.0001*	25.41 (9.60)	*0.0488*
ECM/BCM- Index (I)	1.107 (0.267)	1.320 (0.54)	*0.035*	1.245 (0.52)	*0.0148*	1.188 (0.45)	0.1121
%-cell fraction(I)	48.13 (5.55)	44.74 (7.57)	*0.0033*	45.79 (7.63)	*0.0019*	47.07 (7.14)	0.2366
Body fat abs. (I)	21.20 (9.94)	19.33(10.76)	*0.0410*	18.79 (9.14)	*0.0103*	21.00 (12.79)	*0.0007*
Body fat rel. (I)	27.23 (9.94)	25.29(12.12)	*0.0074*	25.22 (9.75)	*0.0022*	24.95 (9.54)	*0.0435*

Significant values are in italics.

M = mean, SD = standard deviation, I = intervention group.

**Figure 2:**
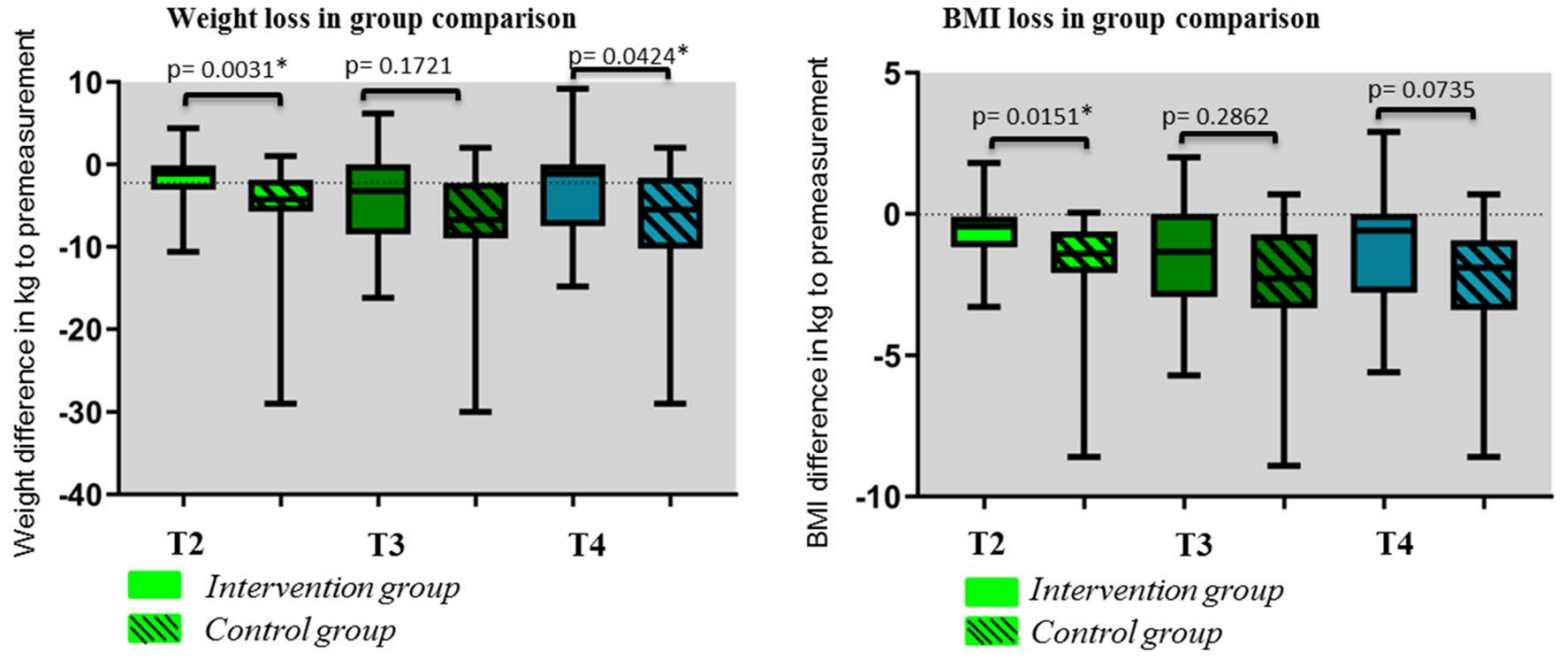
Weight and BMI loss in group comparison at measurement points T2 (before discharge), T3 (3 months after surgery and T4 (6 months after surgery).

Figure 2 illustrates the significantly lower weight loss as well as loss in BMI of the intervention patients compared to the control group at measurement time point T2 as well as the significantly lower weight loss at measurement time point T4. By definition BMI < 18.5 and/or weight loss > 12 months^18^ 6 patients in the intervention group (27.27%) and 11 patients in the control group (45.83%) were malnourished during the 6 months study period.

**Figure 3:**
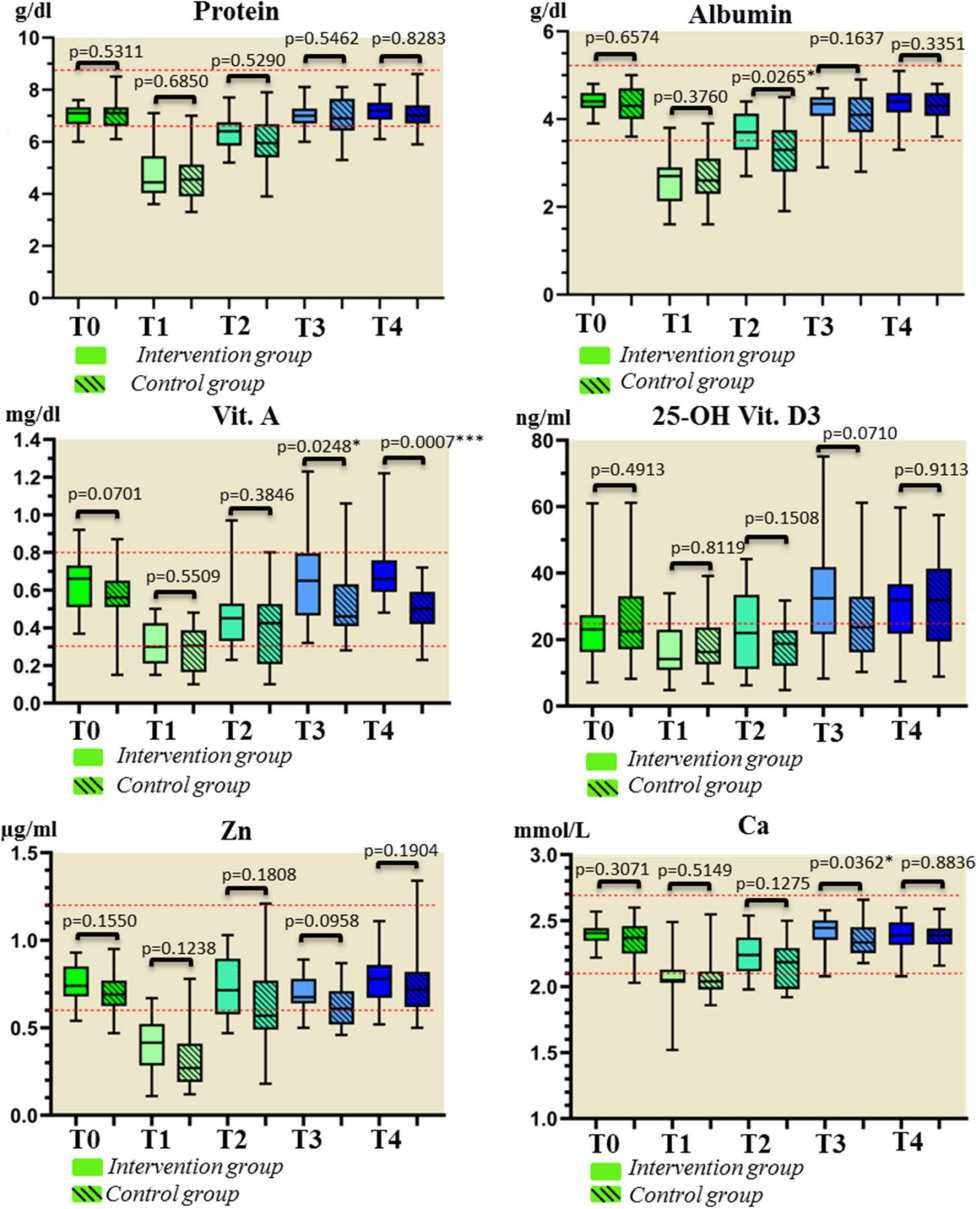
Labor parameters protein, albumin, vitamin A, vitamin D, zinc and calcium at measurement times T0 = before surgery, T1 = first day after surgery, T2 = before discharge, T3 = 3 months postoperative, T4 = 6 months postoperative.

Figure 3 shows the measurements of the laboratory parameters. At measurement time T2 (before discharge), patients in the intervention group achieved significantly more stable measurements of the laboratory parameter albumin. At measurement time T3 (3 months postoperatively), significantly more stable values of the intervention patients were recorded for the parameters vitamin A and calcium, and at measurement time T4 for the parameter vitamin A. Immediately postoperatively, mean values below the reference range were documented for the parameters protein, albumin, vitamin D, zinc, and calcium in both study groups. At T3, the mean values for protein, albumin, vitamin D, and zinc continued to stagnate below the reference range in the control group, whereas in the intervention group, values below the reference range were only recorded for the parameters protein and vitamin D.

Table 3 illustrates, that patients in the intervention group rated their dietary behavior, physical activity, nutrition expertise, attentiveness in nutrition and the impact of the nutritional intervention on physical and psychological recovery significantly higher than patients in the control group. Even six months postoperative, intervention patients evaluated their dietary behavior, physical activity, and nutrition knowledge significantly better than control patients (Table 3). The term dietary/nutritional behavior describes the quality and ability of food intake (e.g. PEG, only liquid food, fast food, balanced diet). Professional nutritional counseling in the intervention group was particularly aimed at improving dietary behavior and the ability of food intake. The responses of the patient-specific aspects were scored on a scale from 0 (low) to 10 (high).

**Table 3:** Patient-specific aspects at measurement times T3 (3 months postop.) and T4 (6 months postop).

		T3			T4		
Specific aspects		I (*n* = 22) M ± SD	C (*n* = 24) M ± SD	*P* Value	I (*n* = 22) M ± SD	C (*n* = 24) M ± SD	*P* Value
Nutritional behavior		4.14 (3.83)	6.71 (3.71)	*0.0348*	3.41(3.74)	5.96(3.83)	*0.0265*
Physical activity		4.09 (1.82)	2.67 (1.52)	*0.0045*	5.00 (2.23)	3.88(2.23)	*0.0283*
Knowledge topic nutrition		6.32 (2.68)	4.17 (2.86)	*0.0128*	6.32 (2.68)	4.33(3.03)	*0.0229*
Attentiveness nutrition		5.68 (1.89)	4.26 (1.98)	*0.0097*	4.96 (2.13)	4.25(1.48)	0.2041
Effect on physical recovery		6.18 (2.13)	5.06 (2.11)	*0.0432*	6.77 (2.51)	6.46(1.89)	0.6358
Effect on mental recovery		6.09 (2.37)	5.00 (2.19)	*0.0332*	6.64 (2.87)	5.96(2.18)	0.3755
Gastrointestinal complaints		1.73 (1.70)	3.50 (2.38)	*0.0062*	2.18 (1.82)	2.42 (2.00)	*>* 0.999
Muscular complaints		2.64 (2.99)	4.38 (3.21)	*0.0448*	2.82 (3.07)	3.67 (3.09)	0.2854
Cardiovascular complaints		2.09 (1.72)	2.00 (1.67)	0.7527	1.82 (1.33)	1.50 (1.06)	0.2221
Breathing complaints		2.96 (2.63)	2.50 (1.98)	0.7471	2.09 (1.61)	2.25 (1.54)	0.5538
Neurological complaints		1.27 (0.94)	2.29 (2.33)	0.0554	1.63 (1.29)	2.04 (2.12)	0.9447

Significant values are in italics.

M = mean, SD = standard deviation, I = intervention group, C = control group.

### Patient-specific aspects

After three months (T3), only 22.72% of the intervention patients were dependent on PEG or liquid food compared to 62.5% of the control patients, so that significantly more intervention patients were able to oralize solid food than patients in the control group (*p*T3 = 0.0380).

At the same measurement time (T3) intervention patients reported significantly fewer gastrointestinal complaints (*p* = 0.0062) and muscular complaints (*p* = 0.0448) than the patients in the intervention group. There were no significant differences in the other complaints.

No harms or unintended effects were registered in the two study groups.

## Discussion

The results of the present study suggest that patients with oral cancer can benefit from an additional postoperative nutritional support.

The measurements of the BIA in the intervention group associate the risk of malnutrition in oral cancer patients and show a stabilization of the nutritional status in the course of the study. We cannot analyze whether stabilization of BIA parameters would have occurred in the control group as well. However, it could be shown for the intervention group.

BIA measurements serve as diagnostic tools for nutritional and supplementary counseling and thus belong to the intervention measures. If BIA measurements had also taken place in the control group, there would have been a risk that the control group could become sensitized to body composition and nutrition at the same time. This could have influenced the comparability of the two groups. For this reason, this study was deliberately omitted from the control group. Intervention patients experienced significantly less postoperative weight loss and reduction in BMI compared to control patients. Measurements of all laboratory parameters showed more stable values in the intervention group compared with the control group, with significant differences only in albumin, vitamin A and calcium. Significant postoperative reproducible nutrient deficiencies in both groups were documented for the parameters protein, albumin, vitamin D, calcium, zinc and iron. The intervention measures in this study had a significant positive effect on postoperative digestive problems and muscular problems as well as on dietary behavior, physical activity and psychological recovery in intervention patients as shown in Table 3.

The significant reduction of weight, BMI, phase angle and ECM/BCM index postoperatively indicates the increased risk of malnutrition and thus also increased morbidity in the first three months after surgery for patients with oral cancer. We did not analyze data on postoperative morbidity, only on postoperative complaints. However, weight, BMI, and especially phase angle and ECM/BCM index are considered generally accepted indicators of increased risk of morbidity^19–22^. The phase angle seems to indicate malnutrition earlier than weight/BMI. These findings correlate with other studies on phase angle, in which values < 4.7 and < 5.0 were attributed to a significantly higher risk of malnutrition^19,20^.

Ravasco et al. even recorded a weight gain in HNC patients with radiation with nutritional advice of 4 kg after three months compared to patients with standard care in combination with nutrient supplementation and control group patients who could not demonstrate any stabilization of weight^14^. An increase in weight in the intervention group could not be determined in the present study. It should be noted, however, that the study patients in Ravasco et al. had already been pretreated with chemotherapy and thus had a lower initial weight^12^, so that weight gain was more likely to be achieved in the course of the study than in patients in this study who were untreated when the preoperative measurements were recorded.

The postoperative significant deficiencies in the laboratory parameters protein, albumin, vitamin D, calcium, zinc and iron can be explained by an increased loss and demand for macro- and micronutrients, which are necessary to support wound healing, the immune response, cell replication/reparation and to reduce postoperative complaints and complications. The more stable measurements of all laboratory parameters in the intervention group compared to the control group seem to be due to the supplementation of protein-rich food and micronutrients. It can be assumed that the significance would have been even more pronounced if the support of the intervention group had already started preoperatively. Severely malnourished patients show delayed wound healing and impaired wound contraction. A malnourished patient has an increased susceptibility to infection and all of this can lead to a prolonged rehabilitation period. When food intake is reduced, this leads to loss of fat, muscle, skin and ultimately bone with subsequent weight loss, and expansion of the extracellular fluid compartment^23^.

In particular, vitamin D and a high-protein diet are associated with a preventive effect on sarcopenia, prolonged wound healing and especially vitamin D is also said to have an anticarcinogenic and anti-inflammatory effect^24–28^. Vitamin D exerts a biological effect on the proliferation and differentiation of myogenic progenitor cells and thus influences muscle regeneration and function and optimizes the rehabilitation measures^25^. Alhambra Expositó et al. did not record any significant changes in serum albumin (*p* = 0.339) and in prealbumin (*p* = 0.797) during nutritional intervention in HNC patients with radiation^16^. The measurements in their study were only taken at two points in time before and after radiotherapy, so that the lack of significance can be justified by the comparatively short measurement period and the preceding chemotherapy as an influence on the measurement parameters. Other nutrition laboratory parameters were not examined.

Through regular consultations and coordinated fat-free and high-fiber dietary measures, significantly fewer digestive complaints and reduction in muscular complaints were registered in the intervention group. Campbell et al. and Albenberg et al. also found a positive correlation between controlled nutrition in the form of a protein- and fiber-rich, low-fat diet and improved intestinal integrity and gut microbiome^29,30^. An improved gut microbiome optimizes the general condition of the patients through increased metabolic activity, endocrine functions and vitamin production as well as support of the immune system^31^.

The significantly better ratings of dietary behavior, physical activity, knowledge about nutrition, mindfulness nutrition and the effect on physical and psychological recovery in the intervention group underline the added value of integrated nutritional counseling in postoperative therapy. Training and motivation to oralize food lead to the intervention patients being able to switch to oral food intake more quickly than the patients in the control group, so the rehabilitation can be accelerated.

The significantly better psychological well-being of the intervention patients during their inpatient stay underlines the positive effect of the integration of a nutrition assistant.

Ravasco et al. documented a significantly better quality of life with the use of a nutritional assistant compared to supplementation alone or to the control group in patients with head and neck cancer^14^. They showed that only the nutritional advice led to a significant improvement in weight, while in the present study it cannot be differentiated whether the nutritional advice or the nutrient supplementation brought about an improvement^14^.

### Limits of the study

The BIA measurements were only performed in the intervention group for a more accurate assessment of the nutrition intervention. An additional implementation of this measurement method in the control group could be considered in order to enable a direct group comparison and a recording of disease-specific reference values. Despite the choice of the comprehensive laboratory profile, the recording of additional parameters such as vitamin C, selenium, prealbumin or other B vitamins would be conceivable in order to determine further interactions and influences on the existing parameters.

Postoperative energy and caloric intake were recorded only in the intervention group, so analyses in group comparison were not possible.

Finally, the postoperative start of supplementation could explain the low number of significances in the laboratory parameters in the group comparison as well as the late significant results of the BIA.

## Conclusion

The results show that patients with oral cancer benefit from an additional postoperative nutritional support. The integration of a nutritional assistant, a protein-rich and high-fiber diet and supplementation for nutrient deficiency lead to less postoperative complaints, better physical and psychological patient status and thus improved rehabilitation. A preoperative start of supplementation, two weeks before surgery, could be considered. In particular, a standardized additional substitution of protein-rich food (e.g. Fortimel Extra 200 ml 1-0-0, high energy protein rich), vitamin D (Decristol 20,000 IE 1xweek), calcium (calcium tablets 1000 mg 1-0-0) and zinc (zinc tablets 40 mg 1-0-0) should be advised for at least three months postoperatively. Preoperative and postoperative screening (BMI, weight loss, if possible: phase angle, lean mass, ECM/BCM index) to assess malnutrition in the clinic and practice is strongly recommended to identify patients at risk and to implement nutritional support measures in collaboration with a professional nutritionist.

## Data Availability

The datasets used and/or analyzed during the current study available from the corresponding author on reasonable request.
